# Co-Targeting Prostate Cancer Epithelium and Bone Stroma by Human Osteonectin-Promoter–Mediated Suicide Gene Therapy Effectively Inhibits Androgen-Independent Prostate Cancer Growth

**DOI:** 10.1371/journal.pone.0153350

**Published:** 2016-04-07

**Authors:** Shian-Ying Sung, Junn-Liang Chang, Kuan-Chou Chen, Shauh-Der Yeh, Yun-Ru Liu, Yen-Hao Su, Chia-Yen Hsueh, Leland W. K. Chung, Chia-Ling Hsieh

**Affiliations:** 1 The Ph.D. Program for Translational Medicine, College of Medical Science and Technology, Taipei Medical University, Taipei, Taiwan; 2 Department of Pathology and Laboratory Medicine, Taoyuan Armed Forces General Hospital, Taoyuan City, Taiwan; 3 Biomedical Engineering Department, Ming Chuan University, Taoyuan City, Taiwan; 4 Graduate Institute of Clinical Medicine, Taipei Medical University, Taipei, Taiwan; 5 Division of Urology, TMU-Shuang Ho Hospital, Ministry of Health and Welfare, New Taipei City, Taiwan; 6 Division of Urology, Taipei Medical University Hospital, Taipei, Taiwan; 7 Joint Biobank, Office of Human Research, Taipei Medical University, Taipei, Taiwan; 8 Division of General Surgery, Department of Surgery, TMU-Shuang Ho Hospital, Ministry of Health and Welfare, New Taipei City, Taiwan; 9 Department of Medicine, Cedars-Sinai Medical Center, Los Angeles, CA, United States of America; Innsbruck Medical University, AUSTRIA

## Abstract

Stromal-epithelial interaction has been shown to promote local tumor growth and distant metastasis. We sought to create a promising gene therapy approach that co-targets cancer and its supporting stromal cells for combating castration-resistant prostate tumors. Herein, we demonstrated that human osteonectin is overexpressed in the prostate cancer epithelium and tumor stroma in comparison with their normal counterpart. We designed a novel human osteonectin promoter (hON-522E) containing positive transcriptional regulatory elements identified in both the promoter and exon 1 region of the human osteonectin gene. *In vitro* reporter assays revealed that the hON-522E promoter is highly active in androgen receptor negative and metastatic prostate cancer and bone stromal cells compared to androgen receptor-positive prostate cancer cells. Moreover, *in vivo* prostate-tumor–promoting activity of the hON-522E promoter was confirmed by intravenous administration of an adenoviral vector containing the hON-522E promoter-driven luciferase gene (Ad-522E-Luc) into mice bearing orthotopic human prostate tumor xenografts. In addition, an adenoviral vector with the hON-522E-promoter–driven herpes simplex virus thymidine kinase gene (Ad-522E-TK) was highly effective against the growth of androgen-independent human prostate cancer PC3M and bone stromal cell line *in vitro* and in pre-established PC3M tumors *in vivo* upon addition of the prodrug ganciclovir. Because of the heterogeneity of human prostate tumors, hON-522E promoter-mediated gene therapy has the potential for the treatment of hormone refractory and bone metastatic prostate cancers.

## Introduction

Prostate cancer is the second-leading cause of cancer-related deaths in both Europe and the United States [1]. Androgen deprivation therapy (ADT) is considered a key treatment as monotherapy or in combination with other regimens. Most patients initially respond to ADT; however, the intrinsic nature of the heterogeneity of tumor cells results in resistance to treatment and progression into highly morbid disease termed castration-resistant prostate cancer (CRPC) within 18–24 months [2]. End-stage CRPC is commonly associated with osseous metastasis, which causes significant mortality and morbidity with the development of severe skeletal complications in affected patients. Recent clinical approaches using agents that target distinct mechanisms of action, including tubulin-binding chemotherapy (cabazitaxel); immunotherapy (sipuleucel-T); CYP-17 inhibition (abiraterone); androgen receptor (AR) blockade (enzalutamide); and radioisotope therapy (radium-223) although have shown promising results in delaying skeletal complications and also improving overall survival [3], the management of patients with metastatic CRPC remains a challenge, with a mean survival time of less than 19 months [4]. Thus, the development of new agents with more effective antitumor activity is crucial for treating metastatic CRPC. In particular, drugs are needed that target hormone-refractory prostate cancer cells regardless of differentiation state, with various levels of androgen receptor (AR) and prostate-specific antigen (PSA) expression.

Past genetic and molecular studies held that tumor cells are heterogeneous and their subsequent metastases are the results of non-random, sequential and multistep selective processes among preexisting cell populations. However, recent studies have evidenced the intricate intercellular communication between stromal and cancer epithelial cells leading to permanent genetic and behavioral changes not only in the epithelial cells but also in cancer-associated stromal cells that drives further genetic and gene expression changes in prostate cancer cells [5, 6]. Through a series of complex, intimate bi-directional communications between prostate cancer and the host stroma, cancer cells gain additional growth and survival advantages and ultimately disseminate to distant organs with lethal effect [7–9]. Thus, co-targeting of both the tumor and its supporting stromal cells can improve therapeutic responses and overall survival of patients with prostate cancer [10–13]. Given that gene therapy has been identified as the preferred treatment for metastatic cancers [14], developing an effective strategy for the delivery and expression of therapeutic genes in the tumor epithelium and adjacent stroma is essential to making such treatment available.

Osteonectin (also known as basement membrane-40 [BM-40] and secreted protein acidic rich in cysteine [SPARC]) is widely distributed in several tissues during development and cellular injury [15] and plays a major role in regulating cell adhesion, proliferation, migration, and tissue remodeling [16]. In the bone microenvironment, osteonectin is the most abundant non-collage matrix protein which is highly expressed early in osteoblastic differentiation and is critical for the maintenance of bone mass [17]. The role of osteonectin in prostate cancer has been identified as a chemoattractant for bone-invasive prostate cancer cells [18–20]. High levels of osteonectin expression have been observed in prostate cancer cell lines derived from metastases and in prostate cancer metastatic foci [21]. In addition, elevated osteonectin levels in primary prostate cancer was associated with the subsequent development of metastasis [22], indicating that prostate cancer cell metastasis to the bone is mediated in part by the osteonectin-mediated promotion of cancer cell migration, protease activity, and invasion. Because osteonectin expression occurs in both tumor epithelial cells and bone cells, the osteonectin promoter could be used to drive a therapeutic gene co-targeting the bone metastatic prostate cancer and its supporting microenvironment, regardless of the basal level of AR and PSA expression.

In this study, we sought to create a promoter–mediated therapeutic agent that co-targets prostate cancer and its surrounding stromal cells. We found that osteonectin was upregulated in prostate cancer epithelial cells and cancer-associated stromal cells compared with their normal counterparts. We designed a novel hON promoter (hON-522E) containing positive transcriptional regulatory elements identified in both the promoter and exon 1 region of the hON gene. We also constructed a replication-defective adenoviral vector bearing a herpes simplex virus thymidine kinase (hsv-TK) gene driven by a highly active 580 bp hON promoter (hON-522E). Treatment with this construct, Ad-522E-TK, in combination with the prodrug ganciclovir (GCV) was found for the first time to kill both androgen-independent prostate cancer and bone stromal cell lines *in vitro* and to inhibit the prostate tumor growth in an xenograft model. Because of the heterogeneity of human prostate tumors, Ad-522E-TK may be applied as an adjunct therapy with other AR-targeting modalities for treatment of hormone refractory and bone metastatic prostate cancers.

## Materials and Methods

### Cell lines and Cell culture

The human prostate cancer cell lines LNCaP, C4-2, C4-2B, PC3, DU145 and PC3M, and a human osteosarcoma cell line MG63 that have been used in our previous studies [6, 23, 24] were maintained in T medium and supplemented with 5% fetal calf serum (FBS). hFOB 1.19 human osteoblast and HS27A human bone marrow stroma cell lines were purchased from ATCC (Manassas, VA, USA) and maintained in a 1:1 mixture of Ham’s F12 Medium/Dulbecco’s Modified Eagle’s Medium and RPMI 1640 medium, respectively, with 10% FBS. The adenovirus packaging 293 cell line (Microbix Biosystems Inc., Toronto, Ontario, Canada) was maintained in Minimal Eagle’s Medium and supplemented with 10% FBS and 2 mM glutamine (Invitrogen, Carlsbad, CA, USA). All cell culture media and reagents were purchased from Invitrogen. All cells were cultured in a 37°C incubator with 5% CO_2_ and were passaged upon reaching 90% confluence.

### Human subject and Laser capture microdissection (LCM)

Experiments with human samples were reviewed and approved by the institutional review board (IRB) at Taipei Medical University (TMU-JIRB 20131253). A prostate tissue microarray (TMA) containing 49 tissue cores representing samples from 40 cases of prostate cancer and 9 matched normal adjacent tissues was obtained from Super Bio Chips (CA4, Seoul, Korea). Frozen human prostate tissue samples were obtained from the TMU Joint Biobank based at the Taipei Medical University and affiliated hospitals from subjects with written informed consent. LCM was used to isolate selectively pure populations of prostate cancer cells and non-neoplastic epithelial cells as well as the stroma adjacent to Gleason grade 3 and grade 4 glands and stroma adjacent to non-malignant glands from frozen sections of prostatectomy specimens derived from four patients. In brief, eight-micron-thick sections of frozen tissue were stained using the Arcturus HistoGene Frozen Section Staining Kit according to the manufacturer’s instructions. Areas of the selected cell populations were microdissected from the sections and collected using the ArcturusXT system and CapSure HS LCM Caps. The settings of the laser were as follows: spot diameter set at 30 μm, power 70 mW, and pulse duration 25 milliseconds. Total RNA from each microdissected sample was extracted using the PicoPure RNA Isolation Kit following the manufacture’s protocol. LCM instrument and all reagents used in the experiment were obtained from Thermo Fisher Scientific Inc. (Madison, WI, USA).

### Real-time RT-PCR

Total RNA was extracted from cells using the High Pure RNA Isolation Kit (Roche, Indianapolis, IN, USA) according to the manufacturer’s instructions. The first-strand complimentary DNA was synthesized using random primers and Moloney murine leukemia virus reverse transcriptase (Invitrogen) and subjected to real-time PCR using the LightCycler 480 with the Light Cycler TaqMan master kit combined with the Universal ProbeLibrary probe (Roche) according to manufacturer instructions. Target genes were amplified using specific primers for hON (forward: 5′-GTGCAGAGGAAACCGAAGAG-3′ and reverse: 5′-TGTTTGCAGTGGTGGTTCTG-3′, probe no. 77), and housekeeping gene, HSPCB (forward: 5′- AGCCTACGTTGCTCACTATTACG-3′ and reverse: 5′- GAAAGGCAAAAGTCTCCACCT-3′, probe no. 55). The relative gene expression of osteonectin in the cell lines is represented as 2^-ΔCT^, with ΔCT determined by subtracting the average housekeeping gene HSPCB threshold cycle from the average target gene value.

### Plasmid construction

All promoter constructs were generated using the TOPO TA cloning system (Invitrogen) and subsequently digested using appropriate restriction sites in the polylinker to allow insertion into the vector pGL3-basic (Promega, Madison, WI, USA) containing the coding region of the firefly luciferase gene. All promoter constructs had the same 5′ end. The spacer between the GGA-boxes 1 and 2 were deleted by recombinant PCR using the following primer sets: 522-N: (5′ACTAGTAGCAGCTTGTCTTGTC3′), spdel-C: (5′CTTCTCCCCTGTCTCTGTCTT3′), and spdel-N: (5′AAGACAGAGACAGGGGAGAAG3′) combined with downstream primers: Intron-C: (5′TACCTCAGTGGCAGGCAGGCAG3′), Exon-C: (5′CAGGCAGGCAGGCGGCAG′), and Hafner-C: (5′GCGCGCTCTCCGGGCAGTCTG3′) to construct hON-522I, hON-522E, and hON-522H, respectively. Genomic DNA was isolated from DU145 cells for the template. All constructs including PCR-generated DNA fragments were confirmed by sequencing.

### DNA transfection and Luciferase assay

Cells were cotransfected with various osteonectin promoter luciferase reporter plasmids and pCMV-βgal (galactosidase) in a 5:1 molar ratio using the lipofectamine 2000 transfection reagent (Invitrogen). After 48 h of incubation, cell extracts were prepared for the luciferase and β-gal activity assessments using the Luciferase Assay System and β-Galactosidase Enzyme Assay System (Promega), respectively, according to manufacturer’s instructions. Relative luciferase activity was calculated as the firefly luciferase relative light units (RLU) divided by the corresponding value for the β-gal activity present in each sample. Three independent experiments were performed in triplicate.

### Design of adenoviral vectors

The Ad-522E-TK and Ad-522E-Luc adenoviral vectors (type 5) were designed and mass-produced according to the established protocol [25]. Briefly, the plasmids p522E-TK and p522E-Luc containing a hON-522E promoter and herpes simplex virus TK gene and luciferase gene, respectively, were constructed by inserting the expression cassette into the E1A deleted region of the Ad5 adenoviral shuttle vector pΔ E1sp1B. A replication-defective recombinant Ad522E-TK adenovirus was generated in the 293 cells by co-transfecting these cells with both the expression shuttle plasmid and a circular Ad genome plasmid (pJM17) using the standard calcium-phosphate precipitation method [26]; Ad-CMV-TK and Ad-CMV-Luc were constructed similarly.

### Thymidine kinase activity assay

TK activity in Ad-522E-TK- and Ad-CMV-TK-infected cell lines was assayed through phosphorylation of [^3^H]GCV [27]. Briefly, the supernatant fraction of crude cell extracts was mixed with an equal volume of TK assay buffer containing 0.2 μCi [^3^H]GCV (Moravek Biochemicals, CA, USA), 3 mM MgCl_2_, 3 mM ATP, 10 μg/μL bovine serum albumin, and 50 mM sodium phosphate buffer (pH 6.5). The reaction mixture was incubated at 37°C for 90 min, transferred to DE-81 discs (Whatman, Hillsboro, OR, USA), air-dried, and washed thoroughly with 50% ethanol. Phosphorylated [^3^H]GCV bound to the discs was determined with a scintillation counter (Beckman Coulter Inc., Schaumburg, IL, USA). Three independent experiments were performed in triplicate.

### *In vitro* cytotoxicity assays

Cells were seeded on 24-well plates at a density of 2 × 10^4^ cells per well. After 24 h, the cells were infected with Ad-522E-TK in the range of 0–100 Multiplicity of Infection (MOI). After a 2 h adsorption, the virus-containing medium was replaced with fresh medium. After 24 h, the cells were incubated in the presence or absence of 10 μg/mL GCV for 5 days followed by a crystal violet staining; subsequently, the relative cell number was assessed at an optical density (OD) of 590 nm after staining. Each experiment was performed in triplicate.

### Animal study

All animal experiments were approved by and complied with the regulations of the Institutional Animal Care and Use Committee (IACUC) of Taipei Medical University (LAC-2013-0047). Six-week-old male athymic nude mice BALB/cAnN.Cg-Foxn1nu/CrlNarl mice were obtained from the National Laboratory Animal Center (Taipei, Taiwan). The animals were kept under standard pathogen-free conditions and cared for according to the criteria outlined in the National Academy of Sciences Guide for the Care and Use of Laboratory Animals. For analysis of the hON-522E promoter activity *in vivo*, 1 × 10^5^ PC3M cells in 10 μL PBS were injected into the ventral prostates of mice. At 5 days after tumor cell injection, tumor-bearing or untreated mice received intravenous administration of 1 × 10^9^ pfu Ad-522E-Luc or Ad-CMV-Luc through the tail vein (n = 5). Mouse organs and prostate tumor xenografts were harvested for the luciferase activity assay 2 days after viral injection. For assessment of Ad-522E-TK combined with GCV-induced inhibition of tumor growth *in vivo*, 5 × 10^5^ PC3M cells in 50 μL PBS were injected subcutaneously into the flanks of the mice. When the tumor became palpable (3–4 mm in diameter), the animals were randomly assigned to 4 experimental groups (n = 8 for each group): group 1, PBS treatment; group 2, GCV only; group 3, Ad-522E-TK combined with PBS; and group 4, Ad-522E-TK combined with GCV. Ad-522E-TK (50 μL; 2 × 10^9^ pfu) in PBS was administrated through intratumoral injection every other day for 3 times. GCV (100 μL) was administrated daily via intraperitoneal injection at a dose of 40 mg/kg body weight for 2 weeks. Bidimensional tumor measurements were performed twice a week with calipers, and the tumor volume was calculated using the simplified formula for a rotational ellipsoid (L × w^2^ × 0.5236). Animals were sacrificed 5 weeks after therapy using CO_2_ for euthanasia, and tumors were excited for histopathologic examination.

### Immunohistochemistry (IHC)

IHC staining was performed using the Novolink Polymer Detection System (Leica Microsystems, Newcastle Upon Tyne, UK) as previously described [28]. Ki-67 protein was detected in tumor xenografts with mouse antihuman Ki-67 monoclonal antibody (1:100; NCL-Ki67-MM1, Leica Biosystems). Apoptosis was evaluated using the Apo-BrdU-IHC In Situ DNA Fragmentation Assay Kit (BioVision, Inc., Milpitas, CA, USA) as described [28]. IHC staining for osteonectin was performed on prostate TMA using an anti–human-osteonectin monoclonal antibody (1:50; NCL-O-NECTIN, Leica Biosystems). Each TMA spot was examined by a pathologist (J.L.C.) using Allred scoring system [29]. The numerical value for overall intensity (intensity score) is based on a 4-point system: 0, 1, 2, and 3 (for none, light, medium, or dark staining). The numerical value for percent stained (proportion score) is determined by a geometric division; no stain = 0; ≤1/100 cells stained = 1; ≤1/10 cells stained = 2; ≤1/3 cells stained = 3, ≤2/3 cells stained = 4; all cells stained = 5. Addition of the two values gives the total Allred score [30].

### Statistical analysis

All data are presented as the mean (standard deviation [SD]) unless otherwise specified. Analysis was performed using the two-tailed Student’s t-test. P < 0.05 was considered significant.

## Results

### Expression of osteonectin in prostate cancer and stromal cells

**Fig 1:**
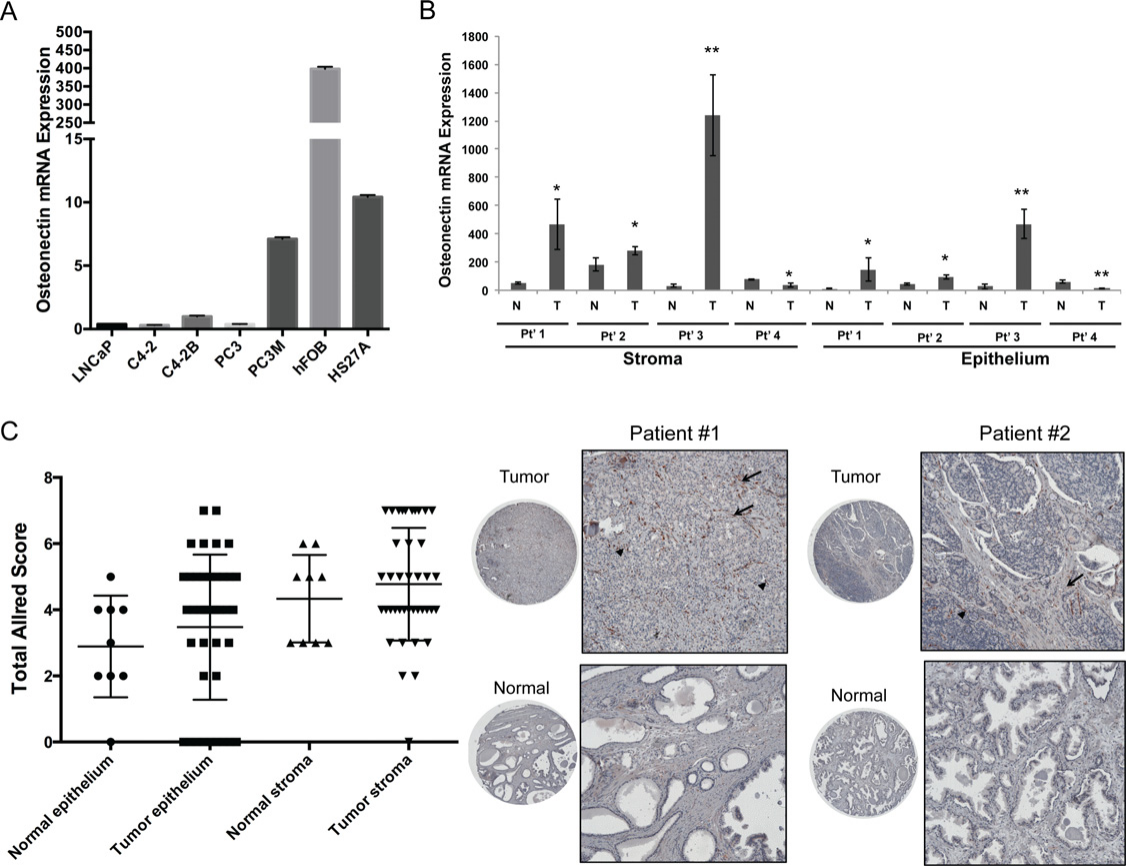
Detection of osteonectin expression in human prostate cancer and prostatic stromal cells. Quantitative RT-PCR analysis of osteonectin mRNA expression in (A) a serial of human prostate cancer and bone stromal (hFOB and HS27A) cell lines and in (B) LCM-isolated prostate epithelial and stromal cells from matched pairs of primary prostate tumor (T) and normal prostate (N) tissues derived from 4 patients (Pt’s 1~4). The relative gene expression of osteonectin was represented as 2^−ΔCT^, with ΔCT determined by subtracting the average housekeeping gene HSPCB threshold cycle from the average target gene value. Data are representative of 3 independent experiments and shown as mean ± SD. *p < 0.05, **p < 0.001 versus normal cells. (C) Scatter plot of IHC staining score for osteonectin in prostate epithelium and stroma in the sections of human prostate tissue microarray containing primary prostate tumor (n = 40) and normal prostate samples (n = 9). Representative images of IHC staining of osteonectin in paired prostate tumor and normal prostate tissues from two individual patients at a magnification of 40 × and 100 × was shown on the right. The arrow and arrowhead indicate positive staining in the stromal and epithelial cells, respectively.

The association of osteonectin expression with human cancer progression was initially evaluated through real-time RT-PCR on the androgen-responsive LNCaP and androgen-insensitive PC3 prostate cancer cell lines. In the series of LNCaP lineage cell lines, the expression of osteonectin correlated with increased bone metastatic potential, in which the hormone refractory and bone metastatic C4-2B expressed a higher level of osteonectin than did its parental androgen-dependent LNCaP and androgen-independent C4-2 cells. Similarly, PC3M, the highly metastatic derivative expressed 18 times higher levels of osteonectin compared with their parental PC3 cells that was originally derived from bone metastases of prostate cancer (Fig 1A). These results revealed a correlation between elevated osteonectin expression and metastatic CRPC progression. As prostate cancer bone metastasis is considered as a microenvironment-driven disease, the higher levels of osteonectin expressed by human bone stromal cells than that of prostate cancer cells was observed using hFOB osteoblast and HS27A bone marrow derived fibroblast cell lines (Fig 1A). To assess the differential expression of osteonectin between the noncancerous and cancerous prostate epithelial cells as well as the normal and cancer-associated stromal cells in same individuals, LCM dissected samples from primary prostate tumors were used. Among the pairs of the normal and malignant prostate tissues, 3 of 4 patients’ samples displayed a significantly increased expression of osteonectin in cancer cells and cancer-adjacent stromal cells in comparison with their normal counterpart; and the other one showed a decreased expression pattern in tumor tissues (Fig 1B).

To further validate the association between the overexpression of osteonectin and prostate carcinogenesis in clinical specimens, immunohistochemical (IHC) analysis was performed on a prostate tissue microarray containing 40 primary prostate tumors and 9 matched normal prostate samples. In each core, immunoreactivity for osteonectin were measured in sections containing normal stroma, normal epithelium, tumor stroma, and tumor epithelium. Although these differences were not statistically significant (P = 0.2654 and 0.4042), the ranks of total Allred score in the overall samples of tumor epithelium and tumor stroma was higher relative to normal epithelium and normal stroma, respectively (Fig 1C). The difference was more significant when only matched pairs of prostate tumor and normal prostate samples was analyzed (P = 0.085 and 0.2165 for epithelial staining and stromal staining, respectively). The lack of statistical significance may be due to the small sample size. In addition, strong staining of osteonectin can be detected in the sample of prostate cancer bone metastases (S1 Fig). The direction of the effect suggests the autocrine and paracrine actions of osteonectin by tumor and tumor microenvironment causing prostate cancer malignancy and metastasis.

### Identification of additional transcriptional regulatory elements in the human osteonectin promoter region

**Fig 2:**
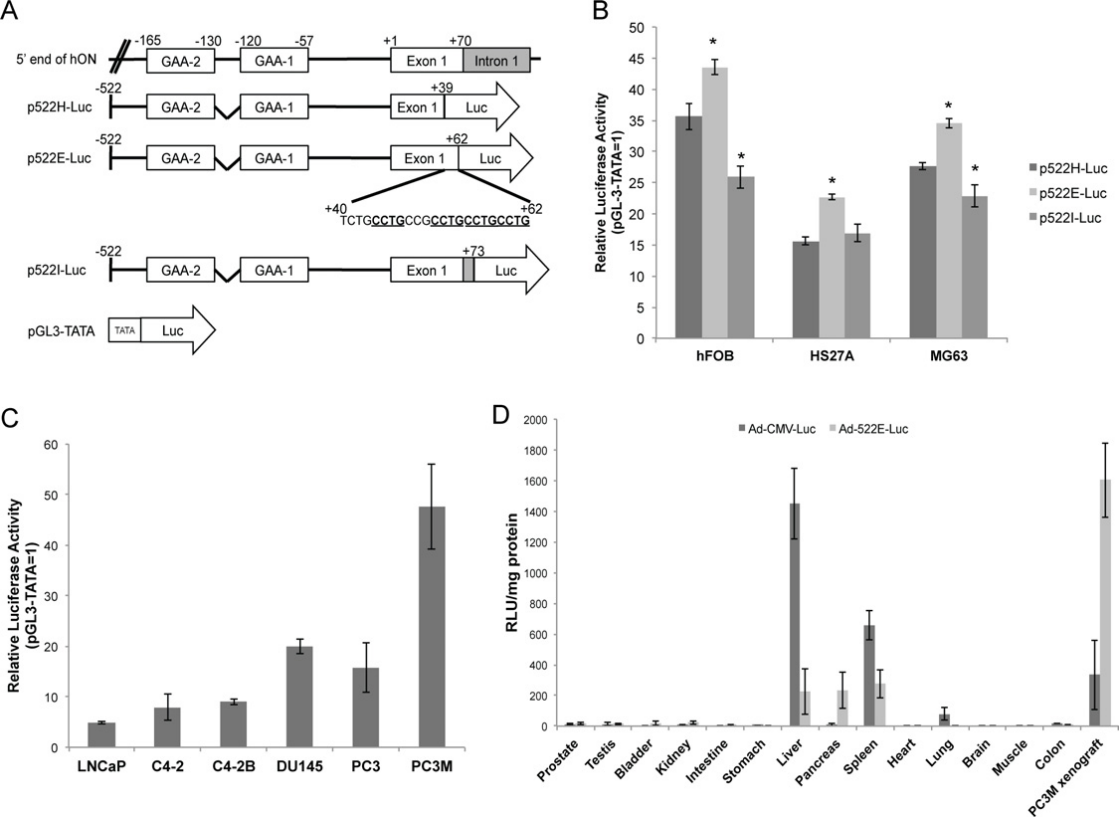
Characterization of osteonectin promoter activity *in vitro* and *in vivo*. (A) Schema of various osteonectin-promoter–driven luciferase constructs. All sequences are numbered relative to +1 (the transcription initiation site). Partial sequence of the osteonectin promoter from bases +40 to +62 containing 4 CCGT motifs (underlined). A modified pGL3 construct (pGL3-TATA) with an artificial TATA box inserted upstream of the luciferase reporter gene was used as a control of basal level promoter activity. (B, C) Comparison of luciferase reporter activity of hON-promoter constructs in human bone stromal cell lines and human prostate cancer cell lines by *in vitro* transfection, and in (D) mouse organs by *in vivo* gene delivery. (B, C) The relative luciferase activity of various constructs was divided by the normalized activity of the empty vector (pGL3-TATA) and expressed as fold-change over control. * p ≤ 0.05 vs. p522H-Luc. (D) Luciferase activities (in relative light units [RLU] per milligram of protein) were determined in the representative organs 2 days after intravenous injection of 1 × 10^9^ pfu of Ad-522E-TK or Ad-CMV-TK into adult mice (n = 5).

The GGA-box 1 in the human osteonectin (hON) promoter region is vital for maximal transcriptional activity, whereas the pyrimidine-rich spacer between GGA-boxes 1 and 2 exerts a downregulatory effect [31]. Comparison of the bovine, mouse, and human osteonectin exon 1 DNA sequences revealed a notable multiple repeat of the sequence CCTG in all species, with a consistent cluster of 7–8 bases upstream from the start of exon 1 [32]. We therefore examined 2 hON-promoter–reporter constructs, p522E-Luc and p522H-Luc, to investigate the role of the CCTG sequence in hON promoter function. The promoter fragment in p522H-Luc is identical to that in pGL2-spdel, which has potential transcriptional activity in human cell lines [31]. p522E-Luc has a 5′ region of hON promoter sequences similar to that of p522H-Luc, but the 3′ end extends to bp + 62, in which 4 CCTG units are included (Fig 2A). Transfection of these constructs and pGL-3-TATA (which serves as a reference of promoter activity) into human bone stromal cell lines, including hFOB, HS27A, and osteosarcoma MG63 showed a marked increase in luciferase activity by p522E-Luc compared with p522H-Luc (Fig 2B). Further 3′ extension of the promoter into bp +73 located at the intron 1 region (p522I-Luc) resulted in decreased luciferase activity, clearly demonstrating that the region between bp +39 and bp +62 is responsible for the additional upregulation of hON promoter activity in human bone cells, whereas the region between bp +63 and bp +73 contains a negative regulatory element.

We next evaluated the transcriptional activity of the hON-522E promoter in various prostate cancer cell lines reflecting different stages of prostate cancer progression. The transfection data showed that hON-522E promoter activity was detected in all cell lines tested. However, comparison of the luciferase activity of these transfected prostate cancer cell lines and those with endogenous osteonectin RNA expression (Fig 1A) revealed that hON-522E promoter activity is relatively higher in AR-negative, more aggressive, and metastatic cell lines, including DU145, PC3 and PC3M cells as compared to AR-expressing LNCaP, C4-2 and C4-2B cell lines (Fig 2C).

To assess the potential utility of the hON-522E promoter in expressing a transgene in a tissue-specific manner *in vivo*, an adenoviral vector containing the hON-522E promoter or the ubiquitous cytomegalovirus (CMV) promoter driving the luciferase reporter gene (Ad-522E-Luc or Ad-CMV-Luc) was administered intravenously to male athymic mice either healthy or carrying orthotopic PC3M tumors. After 2 days of viral administration, major organs, including the liver, lung, kidney, spleen, intestine, heart, brain, colon, testes, prostate, and muscles, and PC3M tumor xenografts were harvested to measure the luciferase expression (Fig 2D). We observed that in the normal organs, Ad-522E-Luc mediated luciferase transgene expression in the liver, spleen, and lung was significantly lower than that of Ad-CMV-Luc, with reduction rates of 16%, 41%, and <1%, respectively. Consistent with a previous study revealing increased expression of osteonectin mRNA in pancreatic ductal epithelial cells [33], luciferase activity transduced by Ad-522E-Luc in the pancreas was 34 times higher than that induced by Ad-CMV-Luc. While the luciferase activity was barely detected in the normal mouse prostate, regardless of Ad vector administration, extremely high luciferase expression by Ad-522E-Luc was observed in PC3M prostate xenografts, with expression 5 times greater than that by Ad-CMV-Luc. These results suggest that the hON-522E promoter is suitable with respect to efficient and selective transgene expression for transcriptional targeting of AR-negative and metastatic prostate cancer cells.

### Evaluation of *in vitro* cytotoxicity of recombinant Ad-522E-TK in prostate cancer and bone stromal cells

**Fig 3:**
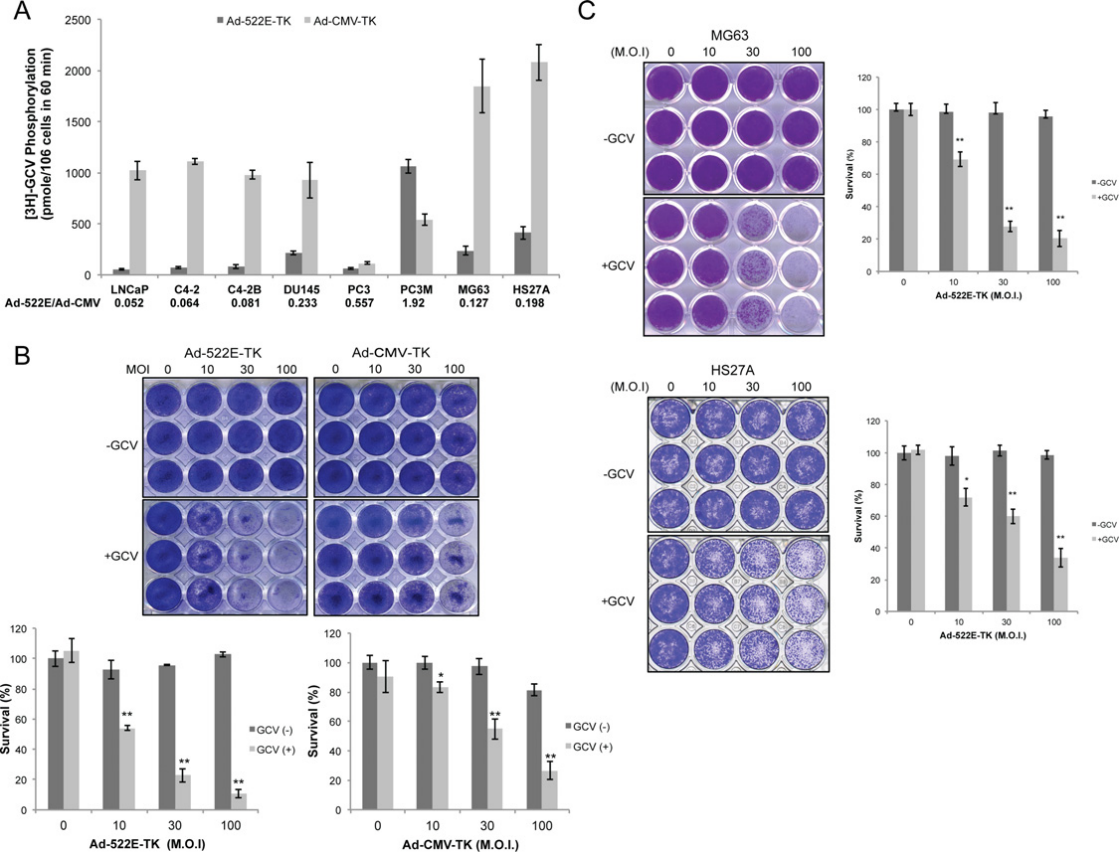
Characterization of Ad-522E-TK-mediated cell death of prostate cancer cells and bone stromal cells *in vitro*. (A). Comparison of TK transgene expression in human prostate cancer cell cells and bone stromal cell lines by Ad-522E-TK and Ad-CMA-TK. Cells were infected with 10 MOI of Ad-522E-TK or Ad-CMV-TK followed by a TK enzyme activity assay. The ratio of TK activities (Ad-522E/Ad-CMV) is indicated. (B) Cytotoxicity of Ad-522E-TK and Ad-CMV-TK combined with GCV in PC3M cells, and (C) Ad-522E-TK in MG63 and HS-27A bone stromal cells. Cells infected with increasing doses of indicated adenoviral vectors were cultured in the presence and absence of GCV for 5 days followed by crystal violet staining. Data are presented as a representative photograph and the percentage of survival by dividing absorbance of treated cells by absorbance of control untreated cells [M.O.I. = 0 and GCV(-)].

To assess the feasibility of hON promoter–directed co-targeting gene therapy for prostate cancer, we designed a replication-deficient adenoviral vector, Ad-522E-TK, carrying the hON-522E promoter–driven herpes simplex virus TK gene. The effectiveness of TK gene delivery in the prostate cancer and bone stromal cell lines was determined using the TK enzymatic activity assay after exposing these cells to Ad-522E-TK and normalized to the external control Ad-CMV-TK for viral infectivity. The cell lines tested, including prostate cancer and bone stromal cell lines, revealed successful TK gene transduction by Ad-522E-TK with an at least two-fold stronger activity in AR-negative prostate cancer cells DU145, PC3 and PC3M as well as bone stromal cells MG63 and HS27A in comparison with AR-positive LNCaP lineage cell lines (Fig 3A), which was similar to the result of luciferase reporter activity by p522E-Luc (Fig 2C). More strikingly, Ad-522E-TK–infected PC3M cells exhibited a higher rather than lower in TK activity than the cells infected with Ad-CMV-TK; this result was consistent with that of Ad-522E-Luc transgene activity in PC3M xenograft tumors (Fig 2D). Upon additional prodrug ganciclovir (GCV) treatment, Ad-522E-TK markedly and dose-dependently decreased the growth of PC3M cells with a higher efficacy than that of Ad-CMV-Luc; Ad-522E-TK or GCV alone exerted no cytotoxic effects on PC3M cells (Fig 3B). In addition, infection of MG63 and HS27A with Ad-522E-TK also induced significant cell death when combined with GCV (Fig 3C). These results demonstrated the ability of Ad-522E-TK to target both prostate cancer and bone stromal cells.

### Antitumor effect of *in vivo* Ad-522E-TK treatment in combination with GCV

**Fig 4:**
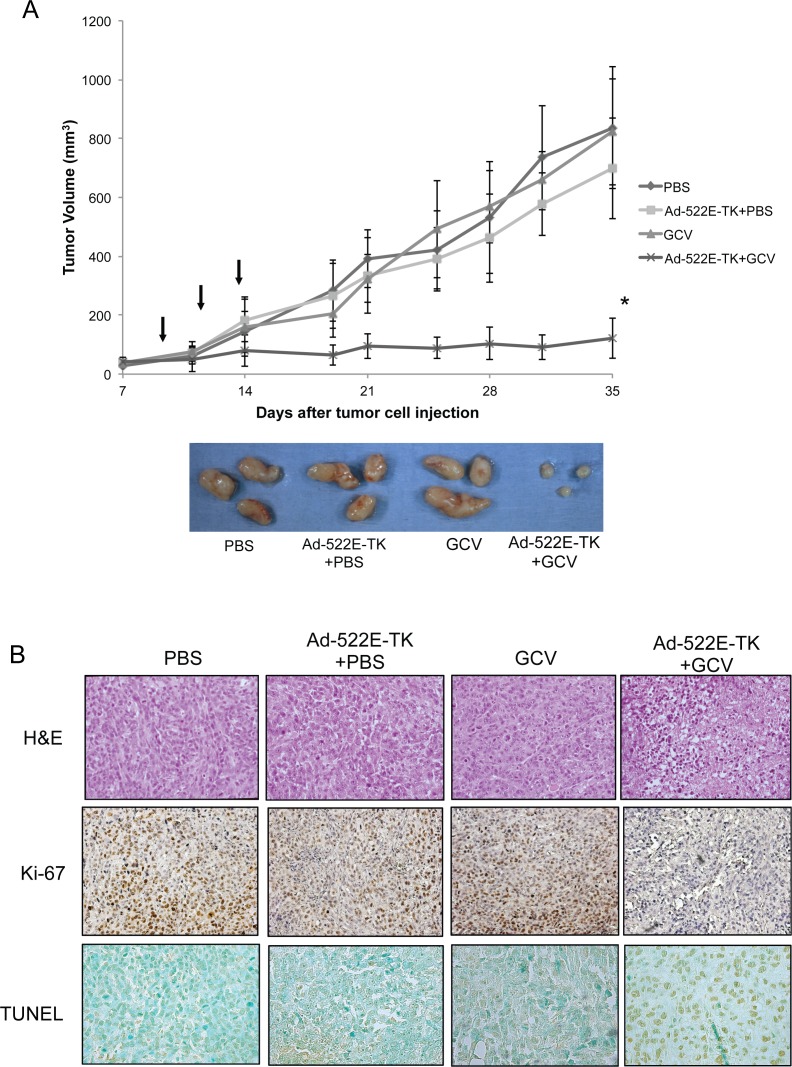
Therapeutic efficacy of Ad-522E-TK combined with GCV on PC3M tumor xenografts in nude mice. (A) Tumor growth kinetics over 5 weeks of follow-up is presented as mean tumor volume ± SD. The arrow indicates the time point of intratumoral injection of Ad-522E-TK (2 × 10^9^ pfu) followed by intraperitoneal injection of GCV (40 mg/kg body weight) daily for 2 weeks. *p < 0.005 compared with the PBS control group. Representative tumors (displayed on the bottom) were excised from 3 of 8 animals in each group at the end of the treatment. (B) Pathological analysis of cytopathic effects (H&E; 200 ×) and detection of cell proliferation (Ki-67; 200 ×) and apoptosis (TUNEL; 400 ×) in tumor tissues of differently treated groups.

To determine the therapeutic efficacy of the hON-522E-promoter–directed gene therapy in the treatment of CRPC human prostate cancer *in vivo*, we evaluated the antitumor effect of Ad-522E-TK combined with GCV in a PC3M subcutaneous xenograft model in nude mice. The PC3M xenograft was observed to be an extremely aggressive tumor that grew to form large tumors (>1 cm diameter) in 5 weeks (Fig 4A). The growth of PC3M tumors was significantly inhibited in animals treated with Ad-522E-TK combined with GCV (p < 0.005). In controls, Ad-522E-TK alone nonsignificantly inhibited tumor growth (p > 0.5), and GCV alone exerted no tumor regression effect as compared to the vehicle (PBS)-treated group. The treated mice did not reveal any gross change in weight. Histological analyses (Fig 4B, 4H & 4E) revealed healthy and packed tumor cells in either PBS-, GCV alone-, or Ad-522E-TK combined with PBS-treated control groups, whereas large necrotic regions were observed in tumors excised from animals treated with the combination ofAd-522E-TK and GCV. In addition, an extensive decrease in proliferative cancer cells (Fig 4B, Ki-67) concomitant with intensely TUNEL-stained apoptotic cells (Fig 4B, TUNEL) within the tumor area confirmed effective cancer cell killing by Ad-522E-TK combined with GCV. Although the current model cannot resemble the microenvironment of bone metastatic prostate cancer, these results as a proof-of-principle study demonstrate the efficacy of Ad-522E-TK plus GCV gene therapy for the treatment of CRPC prostate cancer.

## Discussion

Prostate cancer commonly metastasizes to bony sites, where cells acquire an aggressive, rapidly proliferating, androgen-independent phenotype. The ability of several non-collagenous matrix bone proteins to increase migration and invasion by prostate cancer cell lines has been examined [34], supporting a model in which bone-derived factors attract prostate cancer cells preferentially to such sites. In addition to bone stromal cells such as osteoblasts (hFOB) and bone marrow fibroblasts (HS27A), the results of the present study reveal an apparent upregulation of osteonectin mRNA expression in bone metastatic prostate cancer cells (C4-2B) as compared with their non-bone metastatic sublines (LNCaP and C4-2). Similarly, in the bone-derived prostate cancer PC3 lineage, the highly metastatic variant PC3M cells expressed greater level of osteonectin. This observation is fully consistent with the hypothesis that osseous metastatic prostate cancer cells are osteomimetic, allowing the cells to thrive in bone [35]. Unlike osteocalcin promoter that has been proposed for prostate cancer/bone stroma co-targeting gene therapy based on the conventional osteoblastic reactions demonstrated in experimental models and clinical manifestations of prostate cancer skeletal metastasis [23, 36], elevated osteonectin expression was also observed in prostate cancer epithelium and cancer-associated stroma in primary prostate tumors through RT-PCR and IHC analyses. Therefore, osteonectin-targeted therapy has a broad advantage in blocking paracrine and autocrine events in tumor microenvironment at both prostate and bone sites to prevent and cure bone metastasis in patients.

Although information regarding the expression and potential function of osteonectin in human malignant tumors has increased considerably [37, 38], little is known about the positive and negative regulatory elements in the human osteonectin promoter compared with those in cattle and mice [32, 39, 40]. Two enhancers and a repressor element for the human promoter have been mapped between nucleotides 165 and 130, 51 and 120, and 130 and 121, respectively [31] In the present study, we mapped a region containing a cluster of 4 CCTG repeats between bp +39 and +62 within exon 1 of the human osteonectin gene that is responsible for the additional upregulation in bone stromal cells. The mechanism whereby exon 1 confers tissue-specific gene expression in bone and in particularly in highly bone metastatic prostate cancer PC3M cells is unclear. This positive regulation is probably exerted directly at the transcriptional level through physical interaction with a transcriptional factor that exists in bone cells and prostate cancer cells that have acquired osteomimetic properties. The transcription factor that directly binds to the CCTG repeating sequences is yet to be investigated. In addition, the presence of the repeating CCTG units in the untranslated 5′ mRNA leader probably influences either mRNA translation or stability.

In summary, we have defined and characterized a human osteonectin promoter (hON-522E) that contains only positive transcriptional regulatory elements and is highly active in AR-negative and metastatic prostate cancer cells. When combined with GCV, the recombinant Ad vector Ad-522E-TK was found to effectively induce PC3M cell death *in vitro* and slow the growth of pre-existing PC3M prostate tumors *in vivo*. In this study, only the antitumor effects of Ad-522E-TK through intratumoral administration were examined; the efficacy and safety concerns of systemic use of Ad-522E-TK were not addressed. However, the antitumor effects of osteonectin-promoter–mediated gene therapy for prostate cancer bone metastasis without other organ toxicity might be achieved by understanding the cis- and trans-acting factors in the hON promoter. Because of the heterogeneity of both primary and metastatic prostate tumors, hON-522E-mediated gene therapy may be applied as an adjuvant to AR-targeted therapeutics for treating metastatic CRPC.

## Supporting Information

S1 Fig Immunohistochemical analysis of osteonectin in prostate bone metastasis specimen. Tissue was stained with IgG control antibody (left), or anti-human osteonectin antibody (right); magnification, × 400.
(PDF)
