# Supplementary material for: Co-Targeting Prostate Cancer Epithelium and Bone Stroma by Human Osteonectin-Promoter–Mediated Suicide Gene Therapy Effectively Inhibits Androgen-Independent Prostate Cancer Growth
Source: PLoS One. 2016 Apr 7;11(4):e0153350. doi: 10.1371/journal.pone.0153350 (PMC4824507; doi:10.1371/journal.pone.0153350)
Supplement: S1 Fig — Tissue was stained with IgG control antibody (left), or anti-human osteonectin antibody (right); magnification, × 400. (PDF) [file pone.0153350.s001.pdf]

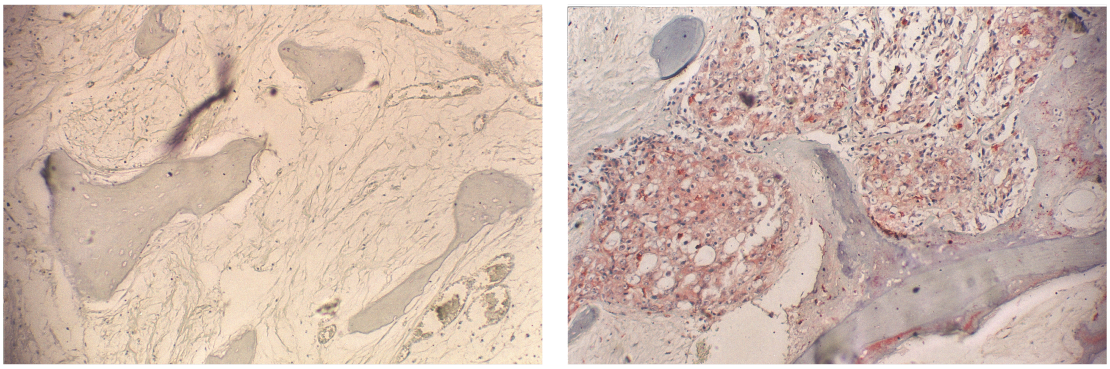

S1 Fig. Immunohistochemical analysis of osteonectin in prostate bone metastasis specimen. Tissue was stained with IgG control antibody (left), or anti-human osteonectin antibody (right); magnification, × 400.
